# Clinical characteristics and prognosis analysis of patients with myocardial infarction with non-obstructive coronary arteries in the Qinghai-Tibet plateau region

**DOI:** 10.3389/fcvm.2025.1590446

**Published:** 2025-07-18

**Authors:** Zixu Fan, Zhiyu Wang, Yinghua Wang, Jianwei Ma, Mingyuan Niu, Min Zhang

**Affiliations:** ^1^ Department of Cardiology, Shanghai Chest Hospital, Shanghai Jiao Tong University School of Medicine, Shanghai, China; ^2^ Department of Laboratory, Shigatse People’s Hospital, Shigatse, China; ^3^ Department of Cardiology, Shigatse People’s Hospital, Shigatse, China

**Keywords:** clinical characteristic, MINOCA, plateau region, ACS, acute myocadial infarction

## Abstract

**Objective:**

To investigate the etiology and clinical characteristics of patients with myocardial infarction with nonobstructive coronary arteries (MINOCA) in the Qinghai-Tibet Plateau region.

**Methods:**

A retrospective analysis was conducted on 82 acute myocardial infarction (AMI) patients who underwent coronary angiography in the Department of Cardiology at Shigatse People's Hospital between December 2020 and December 2021. Patients were divided into two groups based on the results of coronary angiography: the myocardial infarction associated with obstructive coronary artery disease (MI-CAD group, *n* = 67) and the MINOCA group (n = 15). Etiology, medical history, laboratory findings, and in-hospital adverse events were analyzed for the MINOCA group.

**Results:**

Among the 15 MINOCA patients, the primary etiologies included: coronary plaque rupture in 2 cases (13.33%), coronary artery spasm in 2 cases (13.33%), coronary thrombosis or embolism in 1 case (6.67%), type 2 AMI in 1 case (6.67%), unrecognized myocarditis in 3 cases (20%), and other unknown causes in 6 cases. Compared with the MI-CAD group, MINOCA patients had a significantly lower BMI (*p* < 0.05). Laboratory findings revealed that LDL-C, apolipoprotein A, hs-cTnI, and CK-MB levels were significantly lower in the MINOCA group compared to the MI-CAD group (*p* < 0.01). Electrocardiogram results showed lower proportions of T-wave changes and ST-segment elevation in the MINOCA group than in the MI-CAD group (*p* < 0.05). Echocardiography findings indicated that MI-CAD patients were more prone to wall motion abnormalities (*p* < 0.001) and had significantly thicker interventricular septa compared to the MINOCA group (*p* < 0.05).

**Conclusion:**

Due to factors such as hypoxic environments and different lifestyles, MINOCA in the plateau region exhibits characteristics distinct from those observed in low-altitude regions. Enhanced follow-up of these patients is recommended. Further exploration of the mechanisms underlying MINOCA in high-altitude environments is warranted to provide a basis for disease prevention and the development of individualized treatment strategies.

## 1 Introduction

According to the Fourth Universal Definition of Myocardial Infarction (MI), MI is characterized by an elevation of cardiac biomarkers—primarily cardiac troponin (cTn)—exceeding the 99th percentile upper reference limit, with dynamic changes and at least one of the following clinical criteria: (1) Ischemic symptoms; (2) New ischemic electrocardiographic changes (e.g., new ST-T changes or new left bundle branch block); (3) Development of pathological Q waves; (4) Imaging evidence of new loss of viable myocardium or new regional wall motion abnormalities (1). Electrocardiographically, MI is classified as ST-elevation MI (STEMI) or non–ST-elevation MI (NSTEMI). Most cases are due to obstructive coronary artery disease (MI-CAD), defined as ≥50% coronary stenosis on angiography. However, about 6%–8% of MIs occur without significant stenosis (MINOCA, myocardial infarction with nonobstructive coronary arteries), identified by <50% stenosis on angiography (2). Despite earlier beliefs, MINOCA patients have a poor prognosis, with a 12-month all-cause mortality of approximately 3.4% (3). Early recognition and management of MINOCA are essential to improve patient outcomes (2).

The Qinghai-Tibet Plateau, often referred to as the “Roof of the World,” is the highest plateau globally, with an average altitude exceeding 4,500 meters (4). The low oxygen environment at this altitude suppresses aerobic metabolism, resulting in reduced energy production and impaired circulatory function, which limits nutrient and energy delivery to tissues (5). To adapt to hypoxic conditions, the body compensates with an increased heart rate and cardiac output (6). Additionally, high-altitude residents have distinct lifestyles and dietary patterns compared to those at lower altitudes (7). These environmental and behavioral factors may contribute to unique cardiovascular disease profiles among high-altitude residents compared to their low-altitude counterparts.

At present, there is no relevant research on MINOCA patients in the Qinghai-Tibet Plateau. We selected MINOCA patients in Shigatse City (average altitude of about 3,800 meters), and summarized their clinical characteristics, diagnosis,treatment and prognosis. We hope to increase the understanding of MINOCA in the plateau area and provide a reference for optimizing clinical treatment strategies and formulating individualized treatment plans for local patients.

## 2 Materials and methods

### 2.1 General information

A retrospective analysis was performed on 82 patients who were diagnosed with acute myocardial infarction and underwent coronary angiography in Shigatse People's Hospital from December 2020 to December 2021. Inclusion criteria were: (1) meeting the diagnostic criteria for acute myocardial infarction; (2) age >18 years. Exclusion criteria were: (1) type 3, 4, or 5 myocardial infarction; (2) receiving thrombolytic therapy before coronary angiography; (3) pregnant or lactating women; (4) severe liver and kidney diseases; (5) malignant tumors with an expected survival of less than 1 year. The diagnosis of MINOCA was based on the criteria of the fourth edition of the Universal Definition of Myocardial Infarction (1): (1) meeting the diagnostic criteria for acute myocardial infarction; (2) coronary angiography showed non-obstructive coronary artery disease, that is, no obstructive coronary artery disease (i.e., coronary artery stenosis ≥50%) was found in angiography of any possible infarction-related vessels; (3) non-ischemic troponin elevation was excluded, such as systemic factors (renal failure, pulmonary embolism, severe infection, and stroke) and cardiac factors (heart failure). After screening, 15 patients (18.3%) were included in the MINOCA group and 67 patients (81.7%) were included in the MI-CAD group.

### 2.2 Observation indicators

The demographic data and medical history information of all the subjects were collected, including the following: (1) Demographics and Lifestyle Factors: age, sex, smoking history, and body mass index (BMI); (2) Medical History: history of coronary artery disease (CAD), hypertension, diabetes mellitus, cerebrovascular events/transient ischemic attacks (TIA), peripheral arterial disease, hyperlipidemia, and other relevant conditions; (3) Biochemical Parameters (within 24 h of admission): lipid profile: triglycerides, total cholesterol, high-density lipoprotein cholesterol (HDL-C), low-density lipoprotein cholesterol (LDL-C), apolipoprotein A-I, apolipoprotein B, and lipoprotein(a); Cardiac Biomarkers: hypersensitive cardiac troponin I (hs-cTnI) and creatine kinase-MB (CK-MB); Other Blood Tests: blood glucose, glycated hemoglobin (HbA1c), and hypersensitive C-reactive protein (hs-CRP); (4) Imaging and Diagnostic Results: Electrocardiogram (ECG) findings, echocardiographic results; (5) In-hospital adverse events: incidence of infections, deep vein thrombosis (DVT), and major adverse cardiovascular events (MACEs) during hospitalization; (6) All patients were followed for one year. The primary endpoint was the composite of MACEs, including all-cause death, myocardial infarction, ischemic stroke, and heart failure.

### 2.3 Statistical analysis methodology

Data were analyzed using SPSS 26.0 statistical software. For variables conforming to a normal distribution, data were expressed as $\bar{x}\pm s$ (mean ± standard deviation). Group comparisons were performed using *t*-tests. For variables not conforming to a normal distribution, data were presented as *P*_50_ (median) with interquartile ranges (*P*_25_–*P*_75_). Comparisons between groups were conducted using the Wilcoxon rank-sum test. Categorical data were expressed as frequencies (counts and percentages). Relationships between categorical variables were analyzed using the chi-square test (*χ*^2^). *p* < 0.05 was considered statistically significant.

### 2.4 Patient and public involvement

Patients or the public WERE NOT involved in the design, or conduct, or reporting, or dissemination plans of our research.

## 3 Results

### 3.1 Etiology and risk factors of MINOCA

Among all 82 patients, 15 had MINOCA, accounting for 18.3% of all patients, which was significantly higher than previous reports, indicating that patients with myocardial infarction in plateau areas may have characteristics different from those in low-altitude areas. In terms of specific causes, there were 2 cases of coronary artery plaque rupture (13.33%), 2 cases of coronary artery spasm (13.33%), 1 case of coronary artery thrombosis and embolism (6.67%), 1 case of type 2 acute myocardial infarction (6.67%), 3 cases of unrecognized myocarditis (20%), and 6 other cases with no clear cause.

The results (Table 1) showed that all 15 patients in the MINOCA group were male, with a significantly higher proportion than that in the MI-CAD group, but no statistical difference was found (100% vs. 82.1%, *p* = 0.076). At the same time, the BMI of the MINOCA group was lower than that of the MI-CAD group (20.3 kg/m^2^ vs. 24.5 kg/m^2^, *p* = 0.02). There was no statistical difference in age, BMI, smoking history, coronary heart disease history, hypertension history, diabetes history, cerebrovascular accident/TIA history, and hyperlipidemia history between the two groups.

**Table 1:** Comparison of risk factors between MINOCA group and MI-CAD group.

Characteristic	MINOCA group (*n* = 15)	MI-CAD Group (*n* = 67)	*P* value
Male (*n*, %)	15 (100.00)	55 (82.09)	0.076
Age (*y*, $\bar{x}\pm s$)	53.33 ± 12.88	55.00 ± 16.61	0.221
BMI (kg/m^2^, IQR)	20.3 (20.3–24.22)	24.5 (23.0–26.0)	0.020
Smoking history (*n*, %)	7 (46.67)	37 (55.22)	0.548
History of coronary heart disease (*n*, %)	0 (0.00)	4 (5.97)	0.332
History of hypertension (*n*, %)	8 (53.33)	29 (43.28)	0.480
History of diabetes (*n*, %)	1 (6.67)	5 (7.46)	0.915
History of cerebrovascular accident/TIA (*n*, %)	0 (0.00)	1 (1.49)	0.634
History of hyperlipidemia (*n*, %)	0 (0.00)	3 (4.48)	0.404

BMI, body mass index.

### 3.2 Laboratory indicators

The results (Table 2) showed that the low-density lipoprotein cholesterol, apolipoprotein A, hs-cTnI, and CK-MB in the MINOCA group were significantly lower than those in the MI-CAD group (*p* < 0.01), while there were no statistical differences in triglycerides, total cholesterol, HDL-C, apolipoprotein B, lipoprotein (a), blood glucose, glycosylated hemoglobin, and hs -CRP between the two groups.

**Table 2:** Comparison of laboratory parameters between MINOCA group and MI-CAD group.

Variable	MINOCA group (*n* = 15)	MI-CAD Group (*n* = 67)	*P* value
Triglycerides (mmol/L, IQR)	0.95 (0.81–1.30)	1.19 (0.96–1.80)	0.396
Total cholesterol (mmol/L, *x* ± *s*)	3.16 ± 1.61	6.88 ± 13.52	0.65
High-density lipoprotein cholesterol (mmol/L, *x* ± *s*)	0.83 ± 0.44	0.96 ± 0.69	0.568
Low-density lipoprotein cholesterol (mmol/L, IQR)	1.78 (1.60–3.27)	2.94 (2.20–3.54)	0.009
Apolipoprotein A (mmol/L, IQR)	0.81 (0.58–1.01)	0.98 (0.79–1.09)	0.002
Apolipoprotein B (mmol/L, IQR)	0.66 (0.53–0.88)	0.74 (0.60–0.87)	0.427
Lipoprotein (a) (mmol/L, IQR)	332.00 (132.00–403.00)	167.00 (90.00–436.00)	0.496
Blood glucose (mmol/L, IQR)	5.12 (4.72–8.09)	6.18 (5.82–7.41)	0.65
Glycated hemoglobin (%, *x* ± *s*)	5.11 ± 1.39	5.36 ± 1.23	0.643
hs -CRP (mmol/L, IQR)	1.99 (0.50–10.00)	4.30 (0.96–10.00)	0.91
hs-cTnI (ng/ml, IQR)	3.25 (0.43–16.12)	18.61 (3.21–50)	0.007
CK-MB (ng/ml, IQR)	21.90 (3.87–38.39)	68.80 (16.21–248.60)	0.007

hs –CRP, hypersensitive C-reactive protein; hs-cTnI, hypersensitive cardiac troponin I; CK-MB, creatine kinase-MB.

### 3.3 ECG and echocardiogram results

By comparing the ECG characteristics of the two groups of patients (Table 3), we found that the proportion of T wave changes and ST segment elevation in the MINOCA group was lower than that in the MI-CAD group (*p* < 0.05), and there was no statistical difference in the occurrence of atrial fibrillation and ventricular arrhythmia between the two groups. In the analysis of echocardiography (Table 3), we found that patients in the MI-CAD group were more likely to have abnormal ventricular wall contraction activity (*p* < 0.001), and their ventricular septum thickness was significantly higher than that in the MINOCA group (*p* = 0.002). There was no significant difference in the left ventricular ejection fraction (LVEF) between the two groups.

**Table 3:** Comparison of electrocardiogram and echocardiogram results between the MINOCA group and the MI-CAD group.

Variable	MINOCA group (*n* = 15)	MI-CAD Group (*n* = 67)	*P* value
Atrial fibrillation (*n*, %)	1 (6.67)	3 (4.48)	0.722
T wave changes (*n*, %)	4 (26.67)	39 (58.21)	0.027
ST segment elevation (*n*, %)	4 (26.67)	51 (76.12)	<0.001
Ventricular arrhythmia (*n*, %)	0 (0.00)	3 (4.48)	0.404
Abnormal ventricular wall contraction activity (*n*, %)	1 (6.67)	49 (73.13)	<0.001
Interventricular septum thickness (mm, IQR)	9.00 (9.00–9.00)	12.00 (9.00–13.00)	0.002
LVEF (%, IQR)	59.00 (51.00–62.00)	58.00 (48.00–65.00)	0.75

LVEF, left ventricular ejection fraction.

### 3.4 In-hospital adverse events and MACEs

There was no significant statistical difference between the two groups of patients in terms of infection, deep vein thrombosis, heart failure, cardiovascular death and all-cause death (*p* > 0.05, Table 4).

**Table 4:** Comparison of in-hospital adverse events between the MINOCA group and the MI-CAD group.

Events	MINOCA group (*n* = 15)	MI-CAD Group (*n* = 67)	*P* value
Infection (*n*, %)	8 (53.33)	26 (38.80)	0.302
Deep vein thrombosis (*n*, %)	0 (0.00)	2 (3.00)	0.498
Heart failure (*n*, %)	10 (66.67)	42 (62.69)	0.772
Cardiovascular death (*n*, %)	0 (0.00)	1 (1.49)	0.634
All-cause mortality (*n*, %)	0 (0.00)	1 (1.49)	0.634

**Figure 1:**
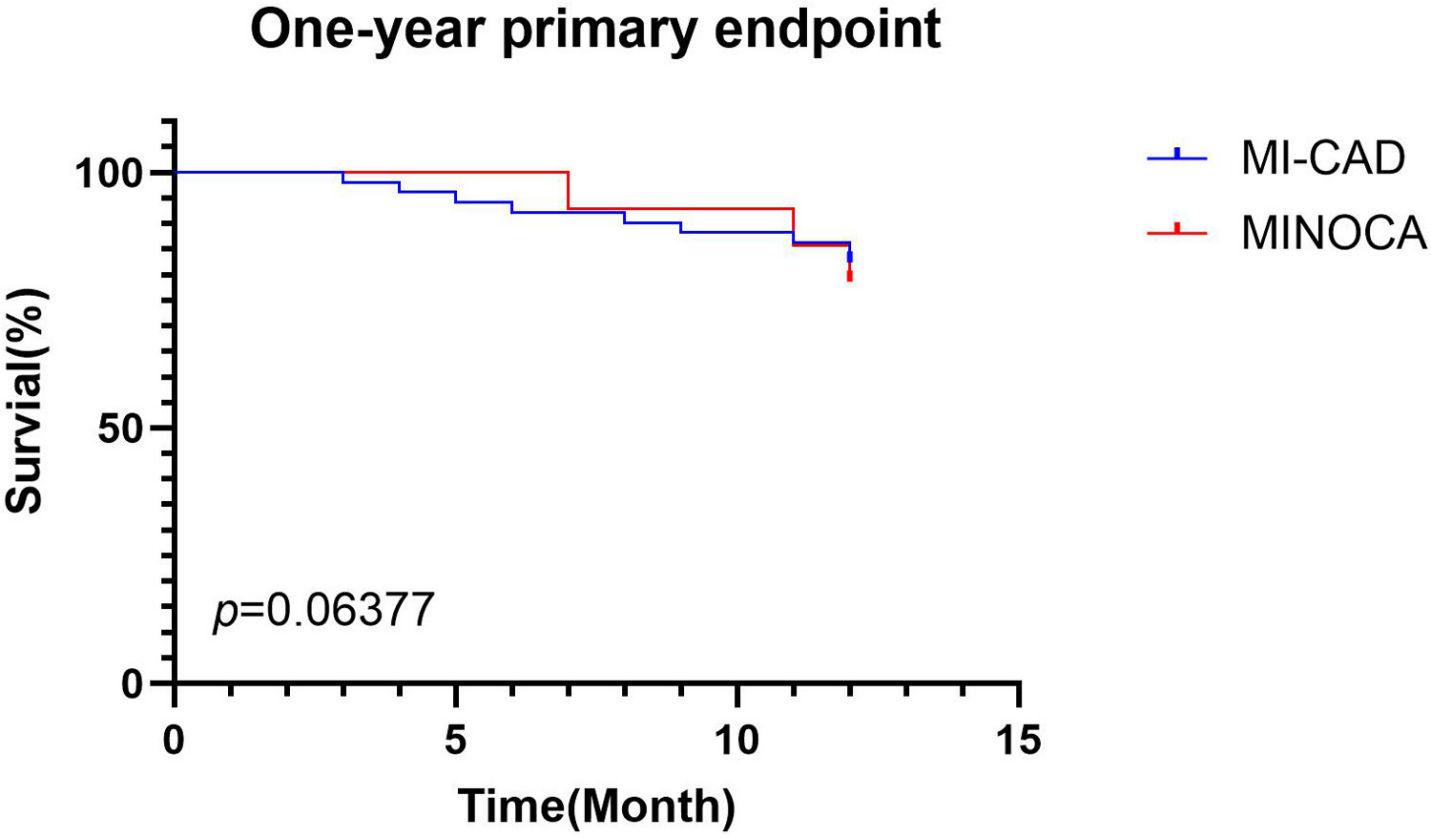
One-year MACEs composite endpoint between the MINOCA group and the MI-CAD group.

At the 1-year follow-up, there was no significant difference in MACE between the MI-CAD group (9 cases) and the MINOCA group (3 cases) (*p* > 0.05, Figure 1).

## 4 Discussion

### 4.1 Incidence of MINOCA

Previous studies have reported that MINOCA accounts for about 6%–8% of acute myocardial infarction cases globally (2, 8, 9), and 5.47% to 9.19% in China (10–12). In this study, MINOCA represented 18.3% of cases, significantly higher than reported rates, indicating that MI in the Qinghai-Tibet Plateau may have unique characteristics. The plateau's chronic hypoxic environment leads to compensatory erythrocytosis and various clinical symptoms known as chronic mountain sickness (CMS) (13). Studies have shown that these patients usually have a higher cardiovascular risk, with significantly increased levels of various inflammatory indicators (such as MIP-1β, IL-8, IFN-γ, etc.) and oxidative stress indicator superoxide dismutase (SOD). At the same time, the patient's microcirculation and macrovascular function will also change due to changes in altitude (14). Whether these factors contribute to the high prevalence of MINOCA in this population warrants further investigation.

### 4.2 Risk factors

Previous studies generally suggest that the proportion of women in MINOCA patients is usually higher than that in MI-CAD patients (1). However, our statistics showed that all 15 MINOCA patients were male, possibly due to the unhealthy lifestyle of local men, exposure to hypoxic environments, and genetic factors. Filippo Zilio et al. explored gender differences in patients with ACS, specifically noting that in many cases, women do not receive timely and adequate diagnosis and treatment (15). In the Qinghai-Tibet Plateau region, women's healthcare seeking rates are even lower, influenced by the social environment and traditional beliefs. Past research reports from China have indicated that among outpatient patients in the Tibet region, there are more males than females, which may also lead to potential research bias (16). Previous studies have found that spontaneous coronary artery dissection (SCAD) and Takotsubo syndrome occur more frequently in women, possibly due to differences in sex hormones and transforming growth factor-β levels. In contrast, myocarditis is more prevalent in men, particularly younger men. Although mortality rates between sexes are similar, further research is needed to clarify the influence of sex on myocarditis outcomes (15, 17).

Consistent with previous studies (18), MINOCA patients had lower BMI and lower levels of blood lipids (LDL-C and apolipoprotein A). In addition, cardiovascular risk factors such as history of hypertension, diabetes, hyperlipidemia, and smoking history vary in different research reports (12, 18). Our statistics found that there were no significant differences between groups in these factors on the plateau. Interestingly, although there was no statistical difference in the age of onset between the two groups of patients, by comparing with previous literature, we found that the average age of patients with myocardial infarction in the Qinghai-Tibet Plateau was significantly lower than that in other areas. Therefore, active health education and cardiovascular screening are needed in plateau areas to enable early detection and intervention, ultimately improving cardiovascular health among residents.

### 4.3 Related inspection results

The results of this study showed that compared with the MI-CAD group, the MINOCA group had lower levels of hs-cTnI and CK-MB, and fewer abnormal ECG manifestations (including T wave changes and ST segment elevation), suggesting that the level of myocardial damage in MINOCA patients was lower. At the same time, the echocardiogram results of the MINOCA group showed fewer abnormal ventricular wall contraction activity, which was consistent with the performance of myocardial markers and ECG. In addition, previous reports have shown (19, 20) that MINOCA patients usually have higher LVEF, but in our study, there was no significant statistical difference in LVEF between the two groups, possibly due to sample size and regional differences. Notably, ventricular septum thickness was significantly lower in the MINOCA group (*p* = 0.002), a finding not been reported in previous studies. There was no significant difference in the history of hypertension between the two groups. Whether this difference is due to the years of hypertension or blood pressure control in the two groups of patients is not yet known, and it deserves further study.

### 4.4 Prognostic analysis

Previous studies have shown that MINOCA patients are more likely to experience in-hospital MACEs compared with MI-CAD patients (11). However, our data showed no significant difference in in-hospital adverse events between the two groups. Possible reasons include: patients had a shorter hospital stay and less chance of complications; the sample size was small and the statistical power was insufficient.

## 5 Study limitations

It is important to note that limited medical resources and low disease awareness among residents of the Qinghai-Tibet Plateau led to delayed medical consultation for chest pain. As a result, our study included only 15 patients in the MINOCA group, which greatly limited the statistical power of our analysis.

On the other hand, other confounding factors may also influence our findings, such as socioeconomic status, racial disparities, dietary patterns, and other high-altitude-related comorbidities. Lower socioeconomic status is linked to higher coronary heart disease mortality (21), making the economic conditions of the Qinghai-Tibet Plateau an important consideration in interpreting our results. Our patient group included both long-term Tibetan residents and tourists; however, due to the small sample size, we could not conduct detailed subgroup analyses to further assess potential differences between these groups.

In patients with MINOCA, cardiac magnetic resonance (CMR) has been shown to have significant diagnostic and prognostic value, particularly in differentiating myocarditis and Takotsubo syndrome, and is highly recommended by current clinical practice guidelines (22–24). A recent study shows that the use of CMR in contemporary cohorts helps distinguish MINOCA from other cardiac conditions, which is important for guiding patient treatment (25). However, due to limitations in local medical resources, CMR cannot be performed. Thus, myocarditis is diagnosed mainly through medical history and clinical presentation; vasospasm is confirmed by symptom relief after nitroglycerin during angiography; and plaque rupture is identified by Intravascular ultrasound (IVUS) or Optical Coherence Tomography (OCT). As understanding of MINOCA deepens, its complexity is increasingly recognized, and its clinical characteristics and management continue to evolve with updated guidelines. Relevant imaging examinations (including CMR, IVUS, OCT, etc.) play an important role in disease differentiation, guiding treatment, and improving prognosis (17, 25).

## 6 Conclusion

MINOCA represents a clinical syndrome caused by diverse etiologies, differing significantly from MI due to coronary artery obstruction in terms of risk factors, pathogenesis, clinical presentation, and prognosis. Recognizing and treating MINOCA remains a challenge in clinical practice. In high-altitude environments, the understanding of this condition is further complicated by limited technical resources, the effects of hypoxia, and unique lifestyle factors. These challenges often result in delayed recognition and diagnosis.

This study retrospectively analyzed MINOCA patients at Shigatse People's Hospital, summarizing their clinical characteristics to provide insights into the diagnosis and treatment of such patients in the Qinghai-Tibet Plateau region. Future efforts should focus on enhanced follow-up of these patients and further exploration of the mechanisms underlying MINOCA in high-altitude environments. These steps are essential for improving disease prevention and developing individualized treatment strategies.

## Data Availability

The raw data supporting the conclusions of this article will be made available by the authors, without undue reservation.

## References

[1] Thygesen K Alpert JS Jaffe AS Chaitman BR Bax JJ Morrow DA Fourth universal definition of myocardial infarction (2018). Circulation. (2018) 138(20):e618–51. 10.1161/CIR.0000000000000617 30571511

[2] Parwani P Kang N Safaeipour M Mamas MA Wei J Gulati M Contemporary diagnosis and management of patients with MINOCA. Curr Cardiol Rep. (2023) 25(6):561–70. 10.1007/s11886-023-01874-x 37067753 PMC10188585

[3] Pasupathy S Lindahl B Litwin P Tavella R Williams MJA Air T Survival in patients with suspected myocardial infarction with nonobstructive coronary arteries: a comprehensive systematic review and meta-analysis from the MINOCA global collaboration. Circ Cardiovasc Qual Outcomes. (2021) 14(11):e007880. 10.1161/CIRCOUTCOMES.121.007880 34784229

[4] Xu S Li S Yang Y Tan J Lou H Jin W A genome-wide search for signals of high-altitude adaptation in Tibetans. Mol Biol Evol. (2011) 28(2):1003–11. 10.1093/molbev/msq277 20961960

[5] Lv J Qi P Bai L Yan X Zhang L. Review of the relationship and underlying mechanisms between the Qinghai-Tibet plateau and host intestinal flora. Front Microbiol. (2022) 13:1055632. 10.3389/fmicb.2022.1055632 36523840 PMC9745141

[6] Moore LG Niermeyer S Zamudio S. Human adaptation to high altitude: regional and life-cycle perspectives. Am J Phys Anthropol. (1998) 27(Suppl ):25–64. <25::AID-AJPA3>3.0.CO;2-L 10.1002/(sici)1096-8644(1998)107:27+<25::aid-ajpa3>3.0.co;2-l 9881522

[7] Lan D Ji W Lin B Chen Y Huang C Xiong X Correlations between gut microbiota community structures of Tibetans and geography. Sci Rep. (2017) 7(1):16982. 10.1038/s41598-017-17194-4 29209019 PMC5717229

[8] Kilic S Aydın G Çoner A Doğan Y Arican Özlük Ö Çelik Y Prevalence and clinical profile of patients with myocardial infarction with non-obstructive coronary arteries in Turkey (MINOCA-TR): a national multi-center, observational study. Anatol J Cardiol. (2020) 23(3):176–82. 10.14744/AnatolJCardiol.2019.46805 32120362 PMC7222639

[9] Dreyer RP Tavella R Curtis JP Wang Y Pauspathy S Messenger J Myocardial infarction with non-obstructive coronary arteries as compared with myocardial infarction and obstructive coronary disease: outcomes in a medicare population. Eur Heart J. (2020) 41(7):870–78. 10.1093/eurheartj/ehz403 31222249 PMC7778433

[10] Mengmeng F Xueping Z Zhiqiang L. Clinical characteristics and prognosis of patients with myocardial infarction with non-obstructive coronary arteries. J Shenyang Med Coll. (2024) 26(2):146–51. https://link.cnki.net/doi/10.16753/j.cnki.1008-2344.2024.02.007

[11] Huang H. Analysis for Clinical Characteristics of Myocardial Infarctionwith Non-Obstructive coronary Arteries. China: Jilin University (2024). Available online at: https://d.wanfangdata.com.cn/thesis/ChhUaGVzaXNOZXdTMjAyNDA5MjAxNTE3MjUSCUQwMzUwNjE4NhoIa3J3YWUybmc%3D

[12] Ruo-chen W Yü-xiang D Jun-bo GE. Differences of clinical characteristics between non-obstructive and obstructive coronary myocardial infarction. Chin J Clin Med. (2021) 28(4):635–39. 10.12025/j.issn.1008-6358.2021.20211231

[13] Corante N Anza-Ramírez C Figueroa-Mujíca R Macarlupú JL Vizcardo-Galindo G Bilo G Excessive erythrocytosis and cardiovascular risk in andean highlanders. High Alt Med Biol. (2018) 19(3):221–31. 10.1089/ham.2017.0123 29782186 PMC6157350

[14] Savina Y Pichon AP Lemaire L Howe CA Ulliel-Roche M Skinner S Micro- and macrovascular function in the highest city in the world: a cross sectional study. Lancet Reg Health Am. (2024) 38:100887. 10.1016/j.lana.2024.100887 39381083 PMC11459627

[15] Zilio F Musella F Ceriello L Ciliberti G Pavan D Manes MT Sex differences in patients presenting with acute coronary syndrome: a state-of-the-art review. Curr Probl Cardiol. (2024) 49(5):102486. 10.1016/j.cpcardiol.2024.102486 38428554

[16] Liu JW Zhang XX. Analysis of outpatient patient situation at a certain hospital in Tibet from 1997 to 2002. Chin J Public Health. (2004) 20(2):227. 10.3321/j.issn:1001-0580.2004.02.054

[17] Ciliberti G Verdoia M Musella F Ceriello L Scicchitano P Fortuni F MINOCA in men and women: different conditions and a single destiny? Int J Cardiol. (2023) 374:6–07. 10.1016/j.ijcard.2022.12.055 36610551

[18] Pasupathy S Air T Dreyer RP Tavella R Beltrame JF. Systematic review of patients presenting with suspected myocardial infarction and nonobstructive coronary arteries. Circulation. (2015) 131(10):861–70. 10.1161/CIRCULATIONAHA.114.011201 25587100

[19] Yang MD. Etiology and clinical features of myocardial infarction with nonobstructive coronary arteries. Chin J Pract Med. (2021) 48(10):17–21. 10.3760/cma.j.cn115689-20210110-00664

[20] Abdu FA Liu L Mohammed A-Q Luo Y Xu S Auckle R Myocardial infarction with non-obstructive coronary arteries (MINOCA) in Chinese patients: clinical features, treatment and 1 year follow-up. Int J Cardiol. (2019) 287:27–31. 10.1016/j.ijcard.2019.02.036 30826195

[21] Rathod KS Lim P Firoozi S Bogle R Jain AK MacCarthy PA The association of socioeconomic status (SES) with procedural management and mortality after percutaneous coronary intervention (PCI): an observational study from the pan-London PCI (BCIS) registry. J Cardiovasc Dev Dis. (2025) 12(3):96. 10.3390/jcdd12030096 40137094 PMC11943075

[22] Bucciarelli V Bianco F Francesco AD Vitulli P Biasi A Primavera M Characteristics and prognosis of a contemporary cohort with myocardial infarction with non-obstructed coronary arteries (MINOCA) presenting different patterns of late gadolinium enhancements in cardiac magnetic resonance imaging. J Clin Med. (2023) 12(6):2266. 10.3390/jcm12062266 36983267 PMC10051168

[23] Mileva N Paolisso P Gallinoro E Fabbricatore D Munhoz D Bergamaschi L Diagnostic and prognostic role of cardiac magnetic resonance in MINOCA: systematic review and meta-analysis. JACC Cardiovasc Imaging. (2023) 16(3):376–89. 10.1016/j.jcmg.2022.12.029 36889851

[24] Byrne RA Rossello X Coughlan JJ Barbato E Berry C Chieffo A 2023 ESC guidelines for the management of acute coronary syndromes. Eur Heart J. (2023) 44(38):3720–826. 10.1093/eurheartj/ehad191 37622654

[25] Ciliberti G Fortuni F Santucci A Timi A Barnoffi E Coiro S Temporal trends of characteristics and management of patients with suspected MINOCA. Int J Cardiol. (2025) 424:133039. 10.1016/j.ijcard.2025.133039 39914630

